# Glucose Fluctuations during Gestation: An Additional Tool for Monitoring Pregnancy Complicated by Diabetes

**DOI:** 10.1155/2013/279021

**Published:** 2013-11-11

**Authors:** M. G. Dalfrà, N. C. Chilelli, G. Di Cianni, G. Mello, C. Lencioni, S. Biagioni, M. Scalese, G. Sartore, A. Lapolla

**Affiliations:** ^1^Department of Medicine, Unit of Metabolic Diseases, University of Padua, Via Giustiniani no. 2, 35128 Padua, Italy; ^2^Department of Endocrinology & Metabolism, Section of Metabolic Diseases & Diabetes, AOUP Pisa, University of Pisa, Ospedale Cisanello, Via Paradisa no. 2, 56124 Pisa, Italy; ^3^Department of Gynecology, Perinatology and Human Reproduction, University of Florence, Viale Giovan Battista Morgagni no. 85, 50134 Florence, Italy; ^4^CNR, Institute of Clinical Physiology, Via G. Moruzzi no. 1, 56124 Pisa, Italy

## Abstract

Continuous glucose monitoring (CGM) gives a unique insight into magnitude and duration of daily glucose fluctuations. Limited data are available on glucose variability (GV) in pregnancy. We aimed to assess GV in healthy pregnant women and cases of type 1 diabetes mellitus or gestational diabetes (GDM) and its possible association with HbA1c. CGM was performed in 50 pregnant women (20 type 1, 20 GDM, and 10 healthy controls) in all three trimesters of pregnancy. We calculated mean amplitude of glycemic excursions (MAGE), standard deviation (SD), interquartile range (IQR), and continuous overlapping net glycemic action (CONGA), as parameters of GV. The high blood glycemic index (HBGI) and low blood glycemic index (LBGI) were also measured as indicators of hyperhypoglycemic risk. Women with type 1 diabetes showed higher GV, with a 2-fold higher risk of hyperglycemic spikes during the day, than healthy pregnant women or GDM ones. GDM women had only slightly higher GV parameters than healthy controls. HbA1c did not correlate with GV indicators in type 1 diabetes or GDM pregnancies. We provided new evidence of the importance of certain GV indicators in pregnant women with GDM or type 1 diabetes and recommended the use of CGM specifically in these populations.

## 1. Introduction

Recent evidence in the literature suggests that glucose variability, characterized by extreme glucose excursions, may overlap with HbA1c levels in giving rise to a risk of diabetes-related complications [1, 2]. Fluctuating blood glucose levels prompt an increase in free radicals and endothelial dysfunction, which link hyperglycemia with the activation of pathological pathways that lead to tissue damage [3]. Reece and Homko postulated an association between maternal hyperglycemia-induced oxygen free radical overproduction and fetal abnormalities, with the onset of diabetes-related embryopathy [4].

Numerous studies have shown that macrosomia and congenital malformations relate to glycemic control [5–7]. In one study, 48-hour continuous glucose monitoring (CGM) of diurnal glucose profiles in pregnant women with type 1 diabetes proved more sensitive than HbA1c alone in identifying a higher risk of offspring developing congenital malformations [8]. Such studies give the impression that transient hyperglycemic spikes in pregnant patients with diabetes could relate to a higher incidence of fetal overweight, via a mechanism at least partly independent of chronic hyperglycemia. Glucose variability is a factor that has yet to be adequately studied in pregnancies complicated by diabetes, and little is known about its relationship with maternal-fetal outcomes [9].

Modern CGM systems can capture the direction and magnitude of short-lived changes in interstitial glucose levels and are therefore useful for assessing glucose variability more accurately than self-monitoring blood glucose (SMBG) measurements [10]. Indeed, it has already been demonstrated that intermittent blood glucose monitoring underestimates the number of hyperglycemic events, because blood glucose excursions can peak at different times of day [11].

A number of studies have demonstrated the important role of CGM for monitoring diabetes in pregnancy [11–13], but several recent trials have partially debunked the utility of real-time CGM in addition to routine care with SMBG [14, 15]. Very few studies have focused attention on the importance of glucose fluctuations during gestation. Meanwhile, there has been a rapid increase in the number of new glucose variability indicators considered, none of which seem to be particularly reliable [16].

A better understanding of the pattern of blood glucose fluctuations in all three trimesters of pregnancy could make it easier to optimize glycemic control in pregnant women with diabetes.

The aim of this study was therefore to assess glucose variability throughout pregnancy in healthy women and in cases of type 1 diabetes mellitus or GDM, comparing different indicators of variability, and to provide more accurate clinical information along with HbA1c and beyond.

## 2. Subjects

The study enrolled 50 outpatient pregnant women, 20 of them with GDM diagnosed according to the Carpenter and Coustan criteria [17]; 20 with type 1 diabetes mellitus, and 10 healthy controls. The women were enrolled during routine visits at three Italian diabetes clinics (Padua, Pisa, and Florence) between 1 January, 2008, and 30 June, 2008, so they can be assumed to be representative of the general diabetic outpatient population. GDM was diagnosed at 20 ± 7.3 weeks of gestation. A subgroup of the GDM women was diagnosed early, during the first trimester of pregnancy. Exclusion criteria were a diagnosis of type 2 diabetes, a pregestational BMI >35 kg/m^2^, and HbA1c >8% for the type 1 diabetic pregnant women. The study protocol complied with the Helsinki Declaration and was approved by the local ethics committees, and written informed consent was obtained from all subjects before their participation in the study.

All the diabetic women were given standard care in accordance with the recommendations of the American Diabetes Association [18]. After being diagnosed with GDM, women were placed on a diet and trained to monitor their glucose levels at home. These GDM patients were asked to measure their glycemic levels four times a day. Insulin treatment was provided when glucose levels continued to exceed 5.28 mmol/L in fasting conditions, or 7.22 mmol/L an hour after meals, after two weeks of nutritional and medical therapy, as recommended by the American College of Obstetricians and Gynecologists (ACOG) guidelines [19]. The women with type 1 diabetes received dietary counseling and intensive insulin therapy. They were also asked to measure their glucose levels 8–10 times a day and trained to adjust their insulin dosage in the light of their glycemic values and any events that might influence their glycemic control. None of the pregnant women with type 1 diabetes used insulin pump devices.

Demographic, anthropometric, and clinical data, that is, age, prepregnancy body mass index (BMI), and weight gain during pregnancy, were recorded for all participants. Weight and height were measured directly and the women reported their pre-pregnancy body weight. Metabolic control was checked in pregnant women with type 1 diabetes or GDM by testing their HbA1c levels. 

For the statistical analysis, the pregnant women were divided into three groups: women with GDM (Group 1), women with type 1 diabetes (Group 2), and healthy women (Group 3).

## 3. Material and Methods

### 3.1. Study Protocol

All the continuous glucose profiles considered in the present study were collected using the GlucoDay system (A. Menarini Diagnostics, Italy). This device is a microdialytic CGM system that measures glucose concentrations in the interstitial fluid continuously over a 48 h period. Its analytical performance is known, as reported in a study by Kovatchev et al. [20] that compared the numerical and clinical accuracy of four CGM systems. Concerning its numerical accuracy, the mean absolute difference (MAD) of the GlucoDay within the range of euglycemic (70–180 mg/dL) was similar to that of other glucometers (15.66 mg/dL). When clinical accuracy was compared using the continuous glucose-error grid analysis (CG-EGA), the results were comparable for all sensors during euglycemia (95.5% of the GlucoDay readings were in zones A + B of the CG-EGA), while the GlucoDay was considerably more accurate during hypoglycemia (96.2% of readings in zones A + B for an interstitial glucose of  70 mg/dL). On the other hand, when Maran et al. tested the GlucoDay in 70 type 1 and 2 diabetic patients in a previous study, they found that 97% of the CGM values fell within the clinically acceptable A + B zone. In a previous paper by Maran et al., moreover, the percentage bias between the GlucoDay and the venous blood levels was −2.0% in the hypoglycemic range (<70 mg/dL), 6.9% in the euglycemic range (70–180 mg/dL), and 11.2% in the hyperglycemic range (>180 mg/dL), showing a good agreement between interstitial glycemia and blood glucose [21]. 

For a monitoring session, the CGM microdialysis probe was inserted subcutaneously in the woman's abdominal region using an 18-gauge Teflon catheter as a guide. After implantation, the probe was connected to the portable CGM device. The CGM takes measurements every 3 minutes, and the results are stored in the device's memory for up to 48 hours. At the end of the monitoring period, the CGM data can be downloaded onto a computer, calibrated retrospectively (against reference blood glucose measurements), and analyzed.

Our patients and healthy pregnant women were trained to make them familiar with glucose monitoring methods, since none of them had ever used the CGM before. They wore the CGM system for 2 days in each trimester of pregnancy, during which they were asked to keep, to their usual pattern of food intake, any insulin injections (for patients in Group 1 and Group 2) and physical activity.

To calibrate the device, reference samples of venous blood were drawn during the 48-hour monitoring period (2, 12, 24, and 48 hours after starting the session), as suggested by the manufacturer. The venous blood samples were stored in fluoride tubes and plasma glucose concentrations were subsequently measured with a standard laboratory instrument using a glucose oxidase method [22]. HbA1c was measured using a standardized LC method and considering the latest reference intervals for HbA1c [23]. All treatment modalities and protocols for the use of CGM were standardized for the different recruiting centers.

### 3.2. CGM Parameter Calculation

To calculate the CGM parameters for the purposes of this study, we considered all the calibrated glucose profiles recorded in the patients' database over a period of 48 hours.

As far as CGM data analysis is concerned, several different parameters for quantifying glucose variability, the quality of glycemic control, and glycemic risk have been amply described in recent years. In this study, as indexes of glucose variability we considered the mean amplitude of glucose excursion (MAGE), calculated as the arithmetical mean of the differences between consecutive glycemic peaks and nadirs, only including changes of more than 1 SD in the glycemic values [24]. We also considered the standard deviation (SD) and the interquartile range (IQR), calculated as the difference between the 75th and 25th percentiles of the glucose levels. Finally, we recorded the continuous overlapping net glycemic action (CONGA), which represents the SD of the glycemic changes recorded between a specific point on the CGM profile and a point *n* hours earlier (where *n* = 1,2, 3,4,…) [25]; we analyzed the CONGA at 1 hour for the purpose of describing short-term, especially postprandial, glucose variability.

The high blood glycemic index (HBGI) and low blood glycemic index (LBGI) account for the frequency and amplitude of hyperglycemic and hypoglycemic events, respectively, enabling an assessment of the risk of patients encountering adverse glycemic events. The LBGI and HBGI were calculated using complex formulas designed specifically to derive these indicators from CGM data [26]. 

The mean glucose value was also calculated from the CGM data to compare patients' glucose variability with their glycemic control, which is also an indicator of mean daily glycemia.

Among the dozens of existing indicators, we chose those capable of specifically describing rapid glucose fluctuations and short-lived hyperglycemic spikes, some of which have been strongly and positively correlated with biochemical markers of oxidative stress [27], while others accurately define and quantify the time course of hypohyperglycemic fluctuations [28]. We included the CGM indicators obtained in the second trimester in this analysis because this was the period in which more data were available, especially as concerns GDM patients. We chose the HbA1c of the 3rd trimester, which reflects the CGM profile of the previous period.

### 3.3. Statistical Analysis

Continuous variables that had a normal distribution are expressed as mean values, accompanied by their standard deviation. Continuous variables with a nonnormal distribution are expressed as median values accompanied by the corresponding interquartile range (25th and 75th quartiles). Comparisons were drawn between groups using the chi-square test with Yates' correction, the Kruskal-Wallis test, the Mann-Whitney *U*-test, the Friedman test, and ANOVA, as appropriate. Associations between continuous variables are expressed as Pearson's correlations, and a *P* value <0.05 was considered significant. The Statistical Package for the Social Sciences (SPSS rel. 16.0, Chicago, IL, USA) was used for the data analysis.

## 4. Results

There were no adverse events associated with the use of the CGM system and all the women tolerated the probe's insertion and wore the device without difficulty.

The mean age of pregnant women was 36.4 ± 4.4 years for Group 1, 33.5 ± 5.2 for Group 2, and 33.9 ± 5.3 for Group 3. Group 2 had been diabetic for a mean 13.7 ± 5.8 years. The women with diabetes had a higher prepregnancy BMI than the healthy controls (26.1 ± 5.8 kg/m^2^ in Group 1, 26.7 ± 6.2 kg/m^2^ in Group 2, and 21.7 ± 1.8 kg/m^2^ in Group 3, *P* = 0.014). HbA1c values decreased in the 3 trimesters in Group 2 (6.0 ± 0.6, 6.4 ± 0.9, 7.2 ± 1.1%; *P* value 1st versus 2nd trimester = 0.002, *P* value 2nd versus 3rd trimester = 0.018), while in Group 1 they did not differ during pregnancy (5.5 ± 0.4, 5.3 ± 1.1, 5.2 ± 0.6%—determined in the 3 trimesters, resp.).

**Figure 1:**
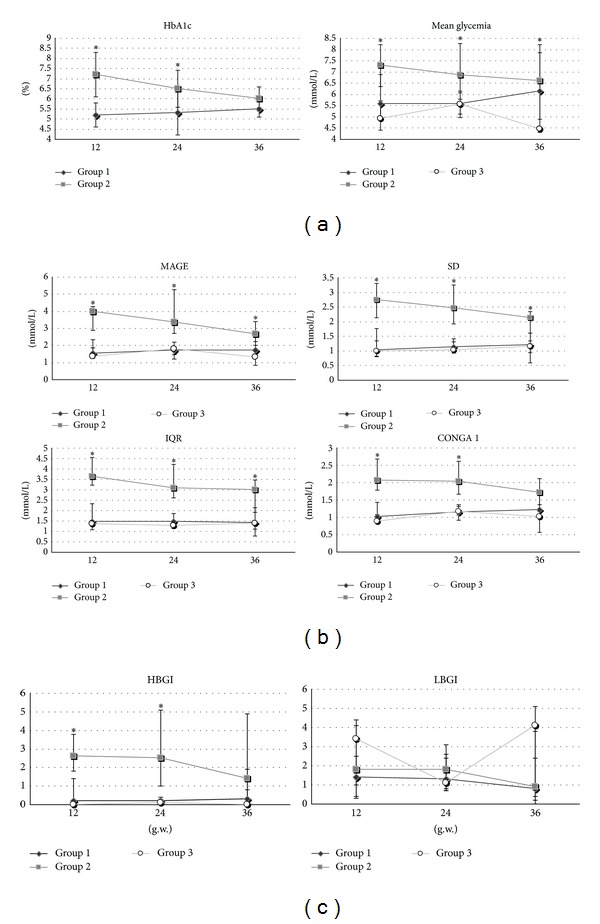
Trend in the three trimesters of pregnancy of indicators of average glycemic control (a), glucose variability (b) and hypohyperglycemic risk (c) in cases of gestational diabetes (Group 1), type 1 diabetes (Group 2) and healthy pregnant women (Group 3). MAGE, mean amplitude of glycemic excursions; SD, standard deviation; IQR, interquartile range; CONGA, continuous overlapping net glycemic action; HBGI, high blood glycemic index; LBGI, low blood glycemic index. Mean value, MAGE, SD, IQR, CONGA 1 are expressed in mg/dL; HbA1c expressed in %; LBGI and HBGI are expressed in classes of risk. *Statistical significance of ANOVA among groups considered for *P* < 0.05.

Figure 1(a) shows the outcome of the analysis of the glucose control indicators in the 3 groups of pregnant women, by trimester of pregnancy. Mean glucose levels (7.29 [range 6.34–8.21], 6.87 [6.07–8.26], 6.62 [6.12–8.81] mmol/L—determined in the 3 trimesters, resp.), and HbA1c levels (7.20 [6.1–8.3], 6.5 [5.6–7.4], 6.0 [5.4–6.6] %) decreased during pregnancy in Group 2 (*P* < 0.05). 

All indicators of glucose variability (Figure 1(b)) were higher in Group 2 than in the other groups in the 1st, 2nd, and 3rd trimesters (*P* < 0.05). In Group 2, MAGE (3.99 [range 2.89–4.27], 3.35 [2.69–5.26], 2.68 [2.22–3.41] mmol/L—determined in the 3 trimesters, resp.), SD (2.75 [2.14–3.30], 2.47 [1.92–3.25], 2.14 [1.61–2.35] mmol/L), CONGA 1 (2.07 [1.78–2.68], 1.71 [1.66–2.62], 2.04 [1.17–2.12] mmol/L), and IQR (3.65 [3.23–4.56], 3.01 [2.6–4.23], 3.08 [1.91–3.47] mmol/L), tended to decrease gradually over the 3 trimesters. On the other hand, for patients in Group 1, there generally tended to be an increase in all glucose variability indicators, for example, MAGE (1.53 [range 1.33–2.33], 1.73 [1.47–1.93], 1.71 [1.34–2.64] mmol/L—determined in the 3 trimesters, resp.), SD (1.03 [0.82–1.76], 1.14 [0.96–1.41], 1.20 [0.94–1.61] mmol/L), CONGA 1 (1.02 [0.92–1.43], 1.15 [0.91–1.32], 1.21 [0.96–1.71] mmol/L), and IQR (1.47 [1.09–2.33], 1.46 [1.39–1.87], 1.43 [1.12–2.13] mmol/L). These differences only reached borderline statistical significance, at most, however.

Finally, Figure 1(c) shows findings on hyperhypoglycemic spikes: HBGI was higher in Group 2 (2.6 [range 1.8–3.8], 2.5 [1.0–5.1], 1.4 [0.3–4.9]—determined in the 3 trimesters, respectively) than in Group 1 (0.2 [0.0–1.4], 0.3 [0.0–0.8], 0.2 [0.1–0.4]) or Group 3. LBGI was higher in Group 3 (3.4 [1.0–4.1], 1.1[0.8–2.6]), 4.1[0.0–5.1]) during the 1st and 3rd trimesters, than in either of the other groups, though the difference did not reach statistical significance (*P* = 0.58, 0.64, 0.87—in the 3 trimesters, resp.). 

As shown in Table 1, the glucose variability indicators generally correlated strongly with one another, with various degrees of statistical significance. The mean glucose values and SD also correlated well with the other indicators for the patients in Group 2 (Table 1(a)), better than for the patients in Group 1 (Table 1(b)). Similar results were found when the first and third trimesters were considered (data not shown). Finally, the HbA1c levels of the patients in Group 2 did not correlate with the glucose variability indicators (Table 1(a)), while borderline associations were identified for a few of these indicators (MAGE and CONGA 1, in particular) in Group 1 (Table 1(b)). 

## 5. Discussion

We assessed glucose variability in the 3 trimesters of normal pregnancy and pregnancy complicated by diabetes, finding a greater glucose variability in pregnant women with type 1 diabetes than in cases of GDM or healthy controls, and the former had a worse interstitial glucose profile than the other two groups.

There is a well-documented relationship between a good glycemic control in pregnant women (be they healthy or diabetic) and fewer congenital malformations and perinatal complications [29, 30]. In healthy pregnant women, an optimal glycemic control is physiological and HbA1c levels are lower than in women who are not pregnant [23, 31]. Since fetal glucose levels depend directly on those of the mother, it follows that mimicking normal glucose patterns in pregnancy complicated by diabetes is the essential goal for the best management of these patients [32].

Although HbA1c remains one of the strongest indicators to predict adverse pregnancy outcomes, in particular in assessing the periconception risk of congenital anomalies [33], there are also contrasting results [34]. Moreover, HbA1c levels do not reflect glucose variability during pregnancy [35], and we still know too little about the role of glucose variability in pregnancies complicated by diabetes [9, 36].

Analyzing the detailed blood sugar profiles obtained by CGM throughout the pregnancy enabled us to strengthen the evidence of a higher glucose variability in healthy pregnant women and in pregnant women with GDM in the second and third trimesters, pointing to the effects of major hormone changes underway in this period, which could exacerbate glycemic instability. This picture could also correlate with physiological changes in the action and secretion of insulin during the third trimester, as previously suggested [37].

Among the novel glucose variability indicators, we also considered those representing the hypo- and hyperglycemic risk. LBGI, which is a specific indicator of hypoglycemic risk, was almost three times higher in our healthy pregnant women than in those with GDM or type 1 diabetes, especially in the first and third trimesters. Our data are consistent with a report from Parretti et al. (although their data only referred to the third trimester), showing that hypoglycemia episodes are quite common in normal pregnancy, especially during the night and early in the morning [37]. This could well be a sign of an increased insulin secretion, associated with hypertrophy and hyperplasia of the *β*-cells, that accompanies the physiological insulin resistance developing during the second and third trimesters of normal pregnancies. This physiological adaptation seems to be impaired in pregnant women with various degrees of glucose intolerance [38].

By measuring the HBGI—a hyperglycemic risk indicator, we showed that pregnant women with type 1 diabetes have at least a 2-fold higher risk of hyperglycemic spikes during the day than healthy pregnant women or those with GDM. This condition exposes pregnant women to a higher risk of fetal congenital malformations and overgrowth during the second and third trimesters [32], so monitoring with HBGI is strongly recommended. As mentioned previously, SMBG only detects a small proportion of these fluctuations [39].

Numerous glucose variability indicators have been developed and there is much confusion about their significance and relationship. In the A1c-Derived Average Glucose (ADAG) study, Borg et al. found that glucose variability indicators generally correlated closely with one another in a large cohort of patients with type 1 and type 2 diabetes, suggesting that these indicators convey much the same information [40]. Our Pearson's analysis confirmed this finding in pregnancies complicated by diabetes. There was a tendency for a correlation between glucose variability and HbA1c, without reaching statistical significance, only in GDM group. This seems to confirm that monitoring HbA1c and glucose variability generates complementary, not overlapping, information [41], and neither of the two alone can ensure a comprehensive assessment of glycemic control. 

The data obtained in this study enable an individualized glucose variability monitoring to be devised for pregnant women with various degrees of glucose intolerance. Given the relevance of assessing glucose variability with a view to optimizing therapeutic strategies and given the higher glucose variability seen in pregnant women with type 1 diabetes, CGM is certainly more appropriate for cases of type 1 diabetes. Among all the indicators and data provided by this electromedical application, the LBGI and HBGI (which reflect major hypo- and hyperglycemic excursions, resp.) seem to be the most useful and sensitive. 

The HBGI could be valuable with a view to improve pregnancy outcomes in pregnant women with GDM too, because of its association with asymmetric intrauterine fetal growth [9]. SD remains the benchmark glucose variability measurement for these patients, however, because it can be obtained by SMBG, which is the most widely used electromedical application. 

The main limitation of our study lies in the small number of patients involved, which may explain why no significant difference in glucose variability emerged from one trimester to another. It is common knowledge that CGM systems measure interstitial glucose. Changes in glucose concentrations in interstitial fluid do not occur at the same time as those in blood; they lag behind, especially when glycemia is changing rapidly, for example, after a meal. The GlucoDay system nonetheless proved highly reliable and reported values very consistent with venous blood glucose measurements [21]. Another limitation of our study concerns the short monitoring period in each trimester, so our conclusions on the glycemic variability indicators must be considered with caution. Finally, although the clinical characteristics of our pregnant women were comparable, our use of multiple recruitment centers and subsequent pooling of the study population is another weakness of this study.

The originality of our work lies in having provided the first CGM-based comparison of pregnant women with and without diabetes in each trimester of pregnancy, with a complete and extensive assessment of their glucose variability, using the most relevant and advanced indicators. In addition, studying the association between glucose variability and HbA1c enabled us to contribute to a better understanding of the roles of these different indicators in glucose control. 

Finally, we provide new evidence of the importance of certain glucose variability indicators in pregnant women with GDM or type 1 diabetes and recommend the use of CGM specifically in these populations. Further studies should concentrate on confirming the benefits of these indicators in daily clinical practice. It would also be a good idea to monitor the same data in a larger cohort of patients all with the same type of diabetes. For the future, now that Murphy et al. have studied the impact of “closed loop” systems in pregnancies complicated by diabetes [42], it will be important to test the benefit of these devices in reducing glucose variability, and thereby hopefully reducing the risk of adverse maternal and fetal outcomes in pregnancies complicated by diabetes.

## Figures and Tables

**Table 1a:** (a)

		MAGE 2	SD 2	IQR 2	CONGA 1-2	LBGI 2	HBGI 2
Mean value 2	Correlation	0.50*	0.62*	0.49*	0.28	−0.61*	0.93*
	*P* value	0.02	<0.01	0.03	0.24	<0.01	<0.001
MAGE 2	Correlation		0.86*	0.74*	0.67*	0.48	0.36
	*P* value		<0.001	<0.001	0.00	0.11	0.13
SD 2	Correlation			0.78*	0.84*	0.29	0.62*
	*P* value			<0.001	<0.001	0.22	<0.01
IQR 2	Correlation				0.54*	0.18	0.59*
	*P* value				0.01	0.43	<0.01
CONGA 1-2	Correlation					−0.06	0.55*
	*P* value					0.81	0.01
LBGI 2	Correlation						−0.50*
	*P* value						0.02
HbA1c 3	Correlation	0.14	0.14	0.09	0.38	−0.22	0.36
	*P* value	0.60	0.62	0.74	0.15	0.41	0.17

**Table 1b:** (b)

		MAGE 2	SD 2	IQR 2	CONGA 1-2	LBGI 2	HBGI 2
Mean value 2	Correlation	0.33	0.62*	0.34	0.23	−0.39	0.09
	*P* value	0.16	<0.01	0.20	0.38	0.13	0.73
MAGE 2	Correlation		0.84*	0.54*	0.54*	0.26	0.69*
	*P* value		<0.001	0.03	0.03	0.33	<0.01
SD 2	Correlation			0.66*	0.29	0.27	0.25
	*P* value			0.01	0.27	0.31	0.34
IQR 2	Correlation				0.73*	−0.26	0.87*
	*P* value				<0.001	0.30	<0.001
CONGA 1-2	Correlation					−0.46*	0.68*
	*P* value					0.05	<0.01
LBGI 2	Correlation						−0.40*
	*P* value						0.04
HbA1c 3	Correlation	0.52	0.45	0.41	0.52	−0.63*	0.42
	*P* value	0.06	0.11	0.15	0.06	0.01	0.14

*Statistical significance considered for *P* < 0.05.
